# Supplementary material for: Dysregulation of erythropoiesis and altered erythroblastic NMDA receptor-mediated calcium influx in Lrfn2-deficient mice
Source: PLoS One. 2021 Jan 22;16(1):e0245624. doi: 10.1371/journal.pone.0245624 (PMC7822338; doi:10.1371/journal.pone.0245624)
Supplement: S1 Fig — (left) BM cell number from a femur. (right) mean cell diameter. WT, n = 14; KO, n = 13 mice at 6–12 M-old male. Live BM cells were analyzed after trypan blue staining using automated cell counter (TC20, Bio-Rad). (PDF) [file pone.0245624.s001.pdf]

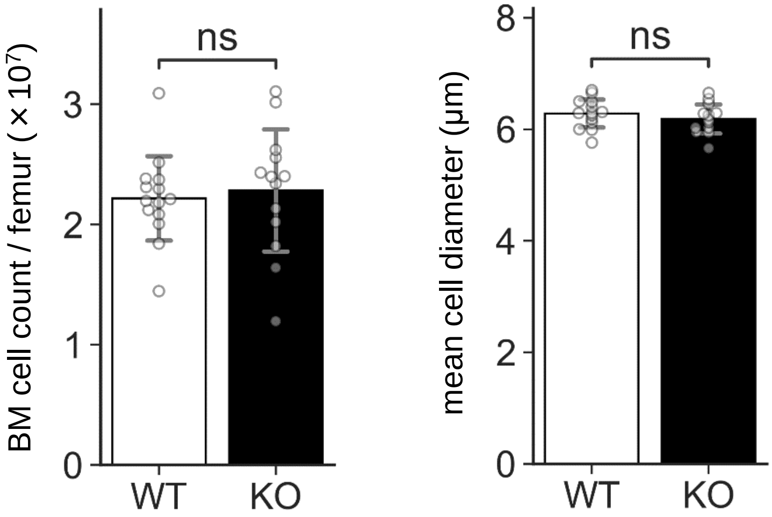

S1 Fig.
Cell number and size of Lrfn2 KO bone marrow.
(left) BM cell number from a femur. (right) mean cell diameter. WT, n = 14; KO, n = 13 mice at 6-12 M-old male. Live BM cells were analyzed after trypan blue staining using automated cell counter (TC20, Bio-Rad).
