# Supplementary material for: Dysregulation of erythropoiesis and altered erythroblastic NMDA receptor-mediated calcium influx in Lrfn2-deficient mice
Source: PLoS One. 2021 Jan 22;16(1):e0245624. doi: 10.1371/journal.pone.0245624 (PMC7822338; doi:10.1371/journal.pone.0245624)
Supplement: S2 Fig — Total RNAs from FACS-fractionated bone marrow cells were subjected to analysis. Cells derived from 5 M-old male mice. Values were normalized to those of 18S ribosomal RNA. WT, n = 3 mice; KO, n = 3 mice. Open bar, WT; closed bar, KO; error bar, SD. Each value from a mouse is indicated by circles. P values were obtained by two tailed t-tests. BM cells were fractionated by BD FACSAria sorter (BD Biosciences). RNA was isolated from the BM cells using TRIzol Reagent (Thermo Fisher). cDNA was synthesized using SuperScript II Reverse Transcriptase (Thermo Fisher). Realtime RT-PCR analysis was carried out using Power SYBR Green PCR Master Mix (Thermo Fisher), QuantStudio 12K Flex Real-Time PCR System (Thermo Fisher). (PDF) [file pone.0245624.s002.pdf]

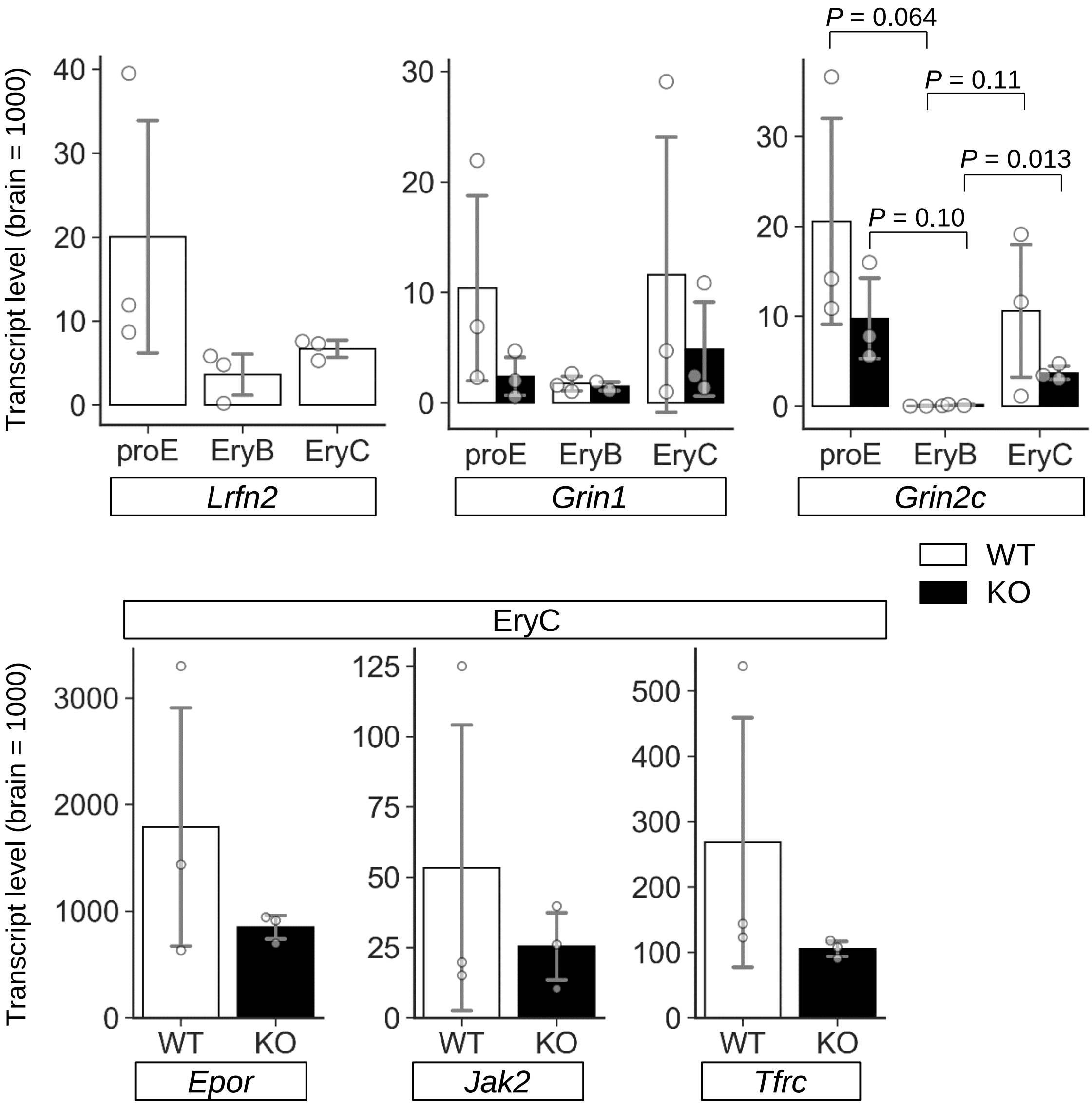

S2 Fig.

Quantitative PCR analysis of mRNA levels. Total RNAs from FACS-fractionated bone marrow cells were subjected for the analysis. Cells derived from 5M-old male mice. Values were normalized by those of 18S ribosomal RNA. WT, *n* = 3 mice; KO, *n* = 3 mice. *Open bar*, WT; *closed bar*, KO; *error bar*, SD. Each value from a mouse is indicated by *circles*. *P* values indicate those obtained by two tailed *t*-tests. BM cells were fractionated by BD FACSAria sorter (BD Biosciences). RNA was isolated from the BM cells with Trizol Reagent (Thermo Fisher). cDNA was synthesized with SuperScript II Reverse Transcriptase (Thermo Fisher). Realtime RT-PCR analysis was carried out using Power SYBR Green PCR Master Mix (Thermo Fisher), QuantStudio 12K Flex Real-Time PCR System (Thermo Fisher).
