# Supplementary material for: Dysregulation of erythropoiesis and altered erythroblastic NMDA receptor-mediated calcium influx in Lrfn2-deficient mice
Source: PLoS One. 2021 Jan 22;16(1):e0245624. doi: 10.1371/journal.pone.0245624 (PMC7822338; doi:10.1371/journal.pone.0245624)
Supplement: S3 Fig — Sections derived from 5–6 M-old male mice. Scale bar, 50 μm. The tissues were fixed by immersion in 4% PFA, 0.1 M sodium phosphate buffer, pH 7.4, overnight at 4 ˚C, dehydrated in a graded series of ethanol, cleared in xylene, and embedded in paraffin blocks for light microscopy. Six-micrometer-thick sections of tissues were cut with a microtome, mounted on slides, and stained with hematoxylin and eosin. Images were taken by BZ-X700 microscope (Keyence). (PDF) [file pone.0245624.s003.pdf]

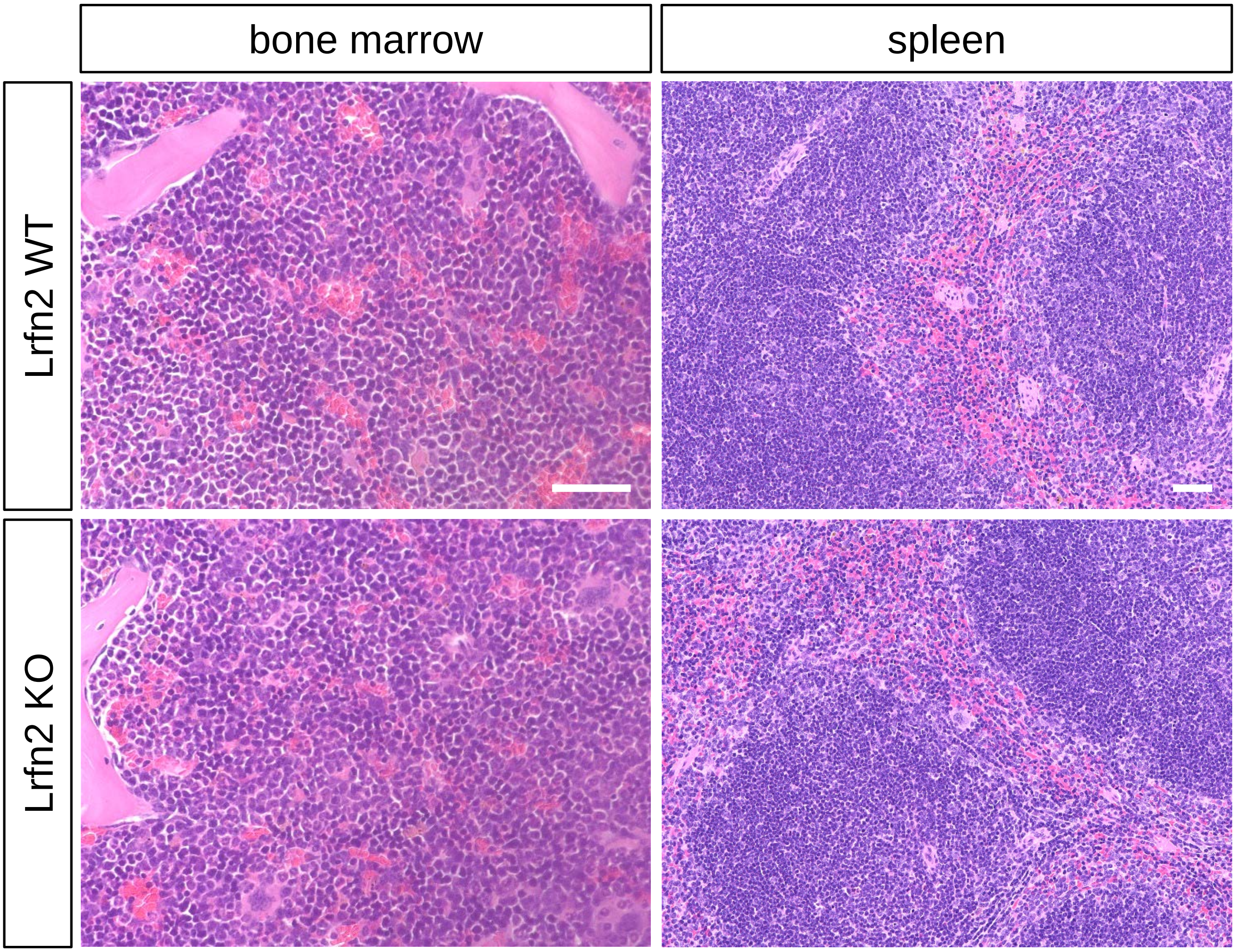

S3 Fig.
Haematoxylin&Eosin staining of bone marrow and spleen sections. Sections derived from 5-6 M-old male mice. Scale bar, 50 μm. The tissues were fixed by immersion in 4%PFA, 0.1 M sodium phosphate buffer, pH 7.4, overnight at 4 ˚C, dehydrated in a graded series of ethanol, cleared in xylene, and embedded in paraffin blocks for light microscopy. Six-micrometer-thick sections of tissues were cut with a microtome, mounted on slides, and stained with haematoxylin and eosin. Images were taken by BZ-X700 microscope (Keyence).
