# Supplementary material for: Dysregulation of erythropoiesis and altered erythroblastic NMDA receptor-mediated calcium influx in Lrfn2-deficient mice
Source: PLoS One. 2021 Jan 22;16(1):e0245624. doi: 10.1371/journal.pone.0245624 (PMC7822338; doi:10.1371/journal.pone.0245624)
Supplement: S1 Table — Complete blood count of male Lrfn2 WT and KO mice aged 3 M (2.0–4.3 M-old; WT, n = 6; KO, n = 7), 6 M (5.8–7.2 M-old; WT, n = 5; KO, n = 6), and 12 M (10.3–13.1 M-old; WT, n = 16; KO, n = 14). Results are indicated as the mean ± SD. Percentages indicate changes in KO mice compared to the mean in WT mice (WT mean = 100%). Italicized values indicate the P values obtained by two-tiered unpaired t-test. Bold letters indicate the percentages and P values with significant differences between WT and KO mice. WBC, leukocyte count; Lymph, lymphocyte count; Mono, monocyte count; Gran, granulocyte count; Lymph%, percentage of lymphocytes in total leukocytes; Mono%, percentage of monocytes in total leukocytes; Gran%, percentage of granulocytes in total leukocytes; RBC, erythrocyte count; HGB, hemoglobin concentration; HCT, hematocrit; MCV, mean corpuscular volume; MCH, mean corpuscular hemoglobin; MCHC, mean corpuscular hemoglobin concentration; RDW; red cell (erythrocyte) distribution width; PLT, platelet count; MPV, mean platelet volume; PDW, platelet distribution width; PCT, plateletcrit. (PDF) [file pone.0245624.s004.pdf]

age	genotype	WBC (10^9/L)		Lymph (10^9/L)		Mon (10^9/L)		Gran (10^9/L)	
3M	WT	12.9±3.9	-8.8%	8.7±3.6	-3.9%	0.35±0.14	-11.4%	3.9±1	-21.4%
	KO	11.9±2.3	*0.56*	8.4±2.9	*0.86*	0.31±0.07	*0.56*	3.2±1.2	*0.29*
6M	WT	10±1.3	**-21.5%**	7.3±0.8	-15%	0.30±0.10	0.0%	2.36±1.43	-52.3%
	KO	8.2±0.9	***0.026***	6.4±1.7	*0.26*	0.30±0.00	*1.00*	1.55±0.16	*0.20*
12M	WT	5.2±2.4	3.4%	3.2±2.2	8.0%	0.15±0.063	-4.8%	1.84±0.89	-3.9%
	KO	5.4±2.1	*0.83*	3.4±1.6	*0.72*	0.143±0.065	*0.76*	1.77±1.03	*0.84*

age	genotype	Lymph%		Mon%		Gran%	
3M	WT	65.2±11.5	4.8%	2.9±0.5	-1.3%	31.9±11.6	-11.4%
	KO	68.5±13.5	*0.65*	2.8±0.3	*0.87*	28.6±13.4	*0.65*
6M	WT	73.2±14.1	5.0%	3.0±0.6	17.1%	23.8±14.2	-23.3%
	KO	77.1±2.5	*0.52*	3.6±0.4	*0.071*	19.3±2.3	*0.46*
12M	WT	59±16.3	8.8%	3.0±0.6	-4.0%	38±16.1	-13.3%
	KO	64.1±13	*0.35*	2.9±0.3	*0.53*	33±12.8	*0.36*

age	genotype	RBC (10^12/L)		HGB (g/L)		HCT %	
3M	WT	9.6±0.7	6.7%	134.2±10.1	5.3%	46.6±3.5	6.2%
	KO	10.2±0.5	*0.071*	141.7±8.3	*0.17*	49.7±2.6	*0.099*
6M	WT	9.5±1.0	**11.3%**	135.6±15.8	**11.2%**	46.2±5.3	**12.0%**
	KO	10.7±0.5	***0.027***	152.7±6.4	***0.038***	52.5±2.2	***0.025***
12M	WT	9.9±0.9	**6.4%**	140.4±15.1	**7.7%**	47.9±4.9	**6.7%**
	KO	10.5±0.6	***0.035***	151.2±9.8	***0.030***	51.1±2.8	***0.038***

age	genotype	MCV (fL)		MCH (pg)		MCHC (g/L)		RDW %	
3M	WT	48.9±0.6	-0.64%	14±0.2	**-1.5%**	287.3±1.8	-1.0%	15.9±0.2	0.008
	KO	48.6±0.6	*0.40*	13.8±0.1	***0.031***	284.4±3.3	*0.081*	16±0.5	*0.6*
6M	WT	48.7±0.7	0.83%	14.2±0.3	-0.28%	293.2±3.8	-1.0%	16.1±0.9	9.1%
	KO	49.1±1.1	*0.49*	14.2±0.2	*0.80*	290.3±2.1	*0.15*	17.7±4.3	*0.43*
12M	WT	48.4±0.8	0.36%	14.1±0.4	1.2%	292.9±5.2	0.85%	15.7±0.4	1.1%
	KO	48.5±1.2	*0.64*	14.3±0.4	*0.26*	295.4±6.3	*0.25*	15.9±0.5	*0.25*

age	genotype	PLT (10^9/L)		MPV (fL)		PDW		PCT %	
3M	WT	795.5±274.1	8.1%	4.2±0.4	-3.2%	16.1±0.2	-0.51%	0.33±0.11	6.3%
	KO	865.3±107.6	*0.55*	4.1±0.2	*0.47*	16±0.2	*0.47*	0.35±0.04	*0.62*
6M	WT	815±352.7	22.9%	4.3±0.3	**-6.5%**	16±0.2	0.54%	0.35±0.1	18.2%
	KO	1058±94.7	*0.14*	4±0.1	***0.036***	16.1±0.1	*0.40*	0.42±0.0	*0.25*
12M	WT	1112±244	0.45%	4.2±0.2	**-4.8%**	16.1±0.2	-0.68%	0.47±0.09	-4.0%
	KO	1117±204	*0.95*	4±0.1	***0.0038***	16±0.1	*0.070*	0.45±0.08	*0.55*

S1 Table.

Hemogram of Lrfn2 KO mice Complete blood count of male Lrfn2 WT and KO mice at 3M (2.0–4.3 M-old; WT, n = 6; KO, n = 7), 6M (5.8–7.2 M-old; WT, n = 5; KO, n = 6), and 12M (10.3–13.1 M-old; WT, n = 16; KO, n = 14). Results are indicated as the mean ± SD. Percentages indicate changes in KO, compared to the WT mean (WT mean = 100%). *Italicized* values indicate the P value obtained by two-tiered unpaired *t*-test. **Bold** letters indicate the percentages and P values with significant differences between WT and KO. WBC, leukocyte count; Lymph, lymphocyte count; Mono, monocyte count; Gran, granulocyte count; Lymph%, percentage of lymphocytes in total leukocytes; Mono%, percentage of monocytes in total leukocytes; Gran%, percentage of granulocytes in total leukocytes; RBC, erythrocyte count; HGB, haemoglobin concentration; HCT, haematocrit; MCV, mean corpuscular volume; MCH, mean corpuscular haemoglobin; MCHC, mean corpuscular haemoglobin concentration; RDW; red cell (erythrocyte) distribution width; PLT, platelet count; MPV, mean platelet volume; PDW, platelet distribution width; PCT, plateletcrit.
