# Supplementary material for: Dysregulation of erythropoiesis and altered erythroblastic NMDA receptor-mediated calcium influx in Lrfn2-deficient mice
Source: PLoS One. 2021 Jan 22;16(1):e0245624. doi: 10.1371/journal.pone.0245624 (PMC7822338; doi:10.1371/journal.pone.0245624)
Supplement: S2 Table — BM cells from 18–22 M-old Lrfn2 WT (n = 8) and KO (n = 7) mice were subjected to CD71/TER119 flow cytometry. Results are indicated as the mean ± SD. Percentages indicate changes in KO mice, compared to the mean in WT mice (WT mean = 100%). Italicized values indicate the P value obtained by two-tiered unpaired t-test. Bold letters indicate the percentages and P values with significant differences between WT and KO. Cell count, numbers of cells out of 10,000 living cells (7AAD-negative); CD71, mean immunofluorescence intensity (arbitrary unit) given by anti-CD71-PE; TER119, mean immunofluorescence intensity (arbitrary unit) given by anti-Ter119-FITC; FSC, forward scatter area; SSC, side scatter area. (PDF) [file pone.0245624.s005.pdf]

	geno-type	Ter119+		pro erythroblast		EryA (early erythroblast)		EryB (mid erythroblast)		EryC (late erythroblast)	
cell count	WT	4091±752	-0.2%	186±52	-8.6%	1050±91	**11.8%**	1610±269	10.3%	1346±551	-22.5%
	KO	4082±324	*0.98*	170±22	*0.46*	1174±59	***0.0089***	1776±164	*0.18*	1043±209	*0.19*
CD71	WT	5208±1536	-3.5%	6509±3530	-11.0%	11448±2641	-8.6%	4868±794	-8.6%	39.6±4.6	**20.6%**
	KO	5028±529	*0.77*	5791±424	*0.60*	10458±995	*0.37*	4451±406	*0.23*	47.7±4.3	***0.0037***
TER119	WT	6285±1369	-1.8%	791±67	4.5%	10348±2732	-6.0%	5371±1302	-5.9%	4209±913	-2.9%
	KO	6170±1053	*0.86*	827±85	*0.38*	9724±1534	*0.60*	5056±992	*0.61*	4086±979	*0.81*
FSC ($\times 10^3$)	WT	53.8±2.9	3.3%	109.2±9.6	-4.3%	105.9±2.4	0.1%	32.0±1.7	0.7%	33.9±1.2	0.3%
	KO	55.6±2.0	*0.20*	104.5±4.5	*0.26*	105.9±2.9	*0.95*	32.2±1.7	*0.80*	34.0±0.7	*0.83*
SSC ($\times 10^3$)	WT	53.5±1.9	-0.3%	107±8.7	-8.3%	78.1±7.2	-1.5%	39.2±2.3	-1.4%	46.9±1.2	0.4%
	KO	53.3±3.1	*0.90*	98±7.9	*0.060*	76.9±7.0	*0.75*	38.7±1.6	*0.60*	47.1±1.5	*0.81*

S2 Table.

Flow cytometry analysis of Lrfn2 KO BM cells. BM cells from 18–22 M-old Lrfn2 WT (*n* = 8) and KO (*n* = 7) mice were subjected to CD71/TER119 flow cytometry. Results are indicated as the mean ± SD. Percentages indicate changes in KO, compared to the WT mean (WT mean = 100%). *Italicized* values indicate the *P* value obtained by two-tiered unpaired *t*-test. **Bold** letters indicate the percentages and *P* values with significant differences between WT and KO. Cell count, numbers of cells out of 10,000 living cells (7AAD-negative); CD71, mean immunofluorescence intensity (arbitrary unit) given by anti-CD71-PE; TER119, mean immunofluorescence intensity (arbitrary unit) given by anti-Ter119-FITC; FSC, forward scatter area; SSC, side scatter area.
